# Genome-wide identification and characterization of long noncoding RNAs during peach (*Prunus persica*) fruit development and ripening

**DOI:** 10.1038/s41598-022-15330-3

**Published:** 2022-06-30

**Authors:** Hui Zhou, Fei Ren, Xiao Wang, Keli Qiu, Yu Sheng, Qingmei Xie, Pei Shi, Jinyun Zhang, Haifa Pan

**Affiliations:** 1 Key Laboratory of Genetic Improvement and Ecophysiology of Horticultural Crops, Institute of Horticulture, Anhui Academy of Agricultural Sciences, Hefei, 230031 China; 2 Institute of Forestry and Pomology, Beijing Academy of Agriculture and Forestry Sciences, Beijing, China; 3 Soil and Fertilizer Research Institute, Anhui Academy of Agricultural Sciences, Hefei, 230031 China; 4 School of Life Science, Anhui Agricultural University, Hefei, China

**Keywords:** Plant development, Non-coding RNAs

## Abstract

LncRNAs represent a class of RNA transcripts of more than 200 nucleotides (nt) in length without discernible protein-coding potential. The expression levels of lncRNAs are significantly affected by stress or developmental cues. Recent studies have shown that lncRNAs participate in fruit development and ripening processes in tomato and strawberry; however, in other fleshy fruits, the association between lncRNAs and fruit ripening remains largely elusive. Here, we constructed 9 ssRNA-Seq libraries from three different peach (*Prunus persica*) fruit developmental stages comprising the first and second exponential stages and the fruit-ripening stage. In total, 1500 confident lncRNAs from 887 loci were obtained according to the bioinformatics analysis. The lncRNAs identified in peach fruits showed distinct characteristics compared with protein-coding mRNAs, including lower expression levels, lower complexity of alternative splicing, shorter isoforms and smaller numbers of exons. Expression analysis identified 575 differentially expressed lncRNAs (DELs) classified into 6 clusters, among which members of Clusters 1, 2, 4 and 5 were putatively associated with fruit development and ripening processes. Quantitative real-time PCR revealed that the DELs indeed had stage-specific expression patterns in peach fruits. GO and KEGG enrichment analysis revealed that DELs might be associated with fruit-ripening-related physiological and metabolic changes, such as flavonoid biosynthesis, fruit texture softening, chlorophyll breakdown and aroma compound accumulation. Finally, the similarity analysis of lncRNAs within different plant species indicated the low sequence conservation of lncRNAs. Our study reports a large number of fruit-expressed lncRNAs and identifies fruit development phase-specific expressed lncRNA members, which highlights their potential functions in fruit development and ripening processes and lays the foundations for future functional research.

## Introduction

Transcriptome studies in eukaryotes including fungi, plants, and animals showed that over 90% of the genome generates a myriad of noncoding RNAs (ncRNAs) including long ncRNAs (lncRNAs)^1,2^. LncRNAs represent a class of RNA transcripts of more than 200 nucleotides (nt) in length without discernible protein-coding potential^3,4^. LncRNAs are enriched not only in the nuclear but also in the cytosol and ribosomal fractions^5^. Some of the lncRNAs located in ribosomal fractions bind with ribosomes, however, these transcripts do not participate in the translation process^5^. Most lncRNAs are transcribed by RNA Pol II either from the sense or the antisense strand in mammals and plants^2,6^, and the lncRNA products of RNA Pol II in plants often show similar features to protein coding mRNAs, such as 5’ capping and 3’ polyadenylation. In plants, lncRNAs are also transcribed by two other plant-specific RNA polymerases, Pol IV and V^7^, and these kinds of lncRNA products are mostly synthesized from transposable elements^8^. Based on their genomic locations, lncRNAs are generally classified into four types: long intergenic noncoding RNAs (lincRNAs), intronic ncRNAs transcribed from introns, sense lncRNAs derived from the same transcriptional direction of their associated genes and antisense lncRNAs derived from the complementary strand^9^.

Compared with protein coding mRNAs, the expression levels of lncRNAs are usually very low^10^. For example, the majority of *Arabidopsis* lincRNAs are expressed at approximately 30–60 fold lower than the associated mRNA levels^11^. Due to the low expression levels, lncRNAs were initially considered transcriptional noise. However, the mechanisms and functions of lncRNAs were preliminarily revealed by systematic functional studies in the last decade. LncRNAs are known to function by regulating the transcription machinery, histone modification and the RNA processing machinery and serving as miRNA sponges^12^. To date, most reports of the functions of lncRNAs in model plants such as *Arabidopsis*, *Oryza sativa* and *Zea mays*, have indicated that they are involved in plant growth and development (e.g., flowering, seed germination, and organ development) and biotic or abiotic stresses (e.g., pathogen or fungi attacks, water or nutrition deficiency, abnormal temperature and light, etc.) response processes^13–15^.

LncRNAs also play important roles in fleshy fruit development and ripening process. In tomato, silencing two lncRNAs, lncRNA1459 and lncRNA1840, retarded the fruit-ripening processes, which indicated the positive regulatory roles of the two members in the fruit-ripening process^16^. Furthermore, lncRNA1459 was proven to be a positive regulator of lycopene, carotenoid and ethylene biosynthesis and numerous ripening-related genes according to the phenotypes of loss-of-function mutants of lncRNA1459 by CRISPR–Cas9 genome editing technology^17^. Moreover, in tomato, 187 lncRNAs were found to be direct targets of the MADS-box transcription factor (TF) RIPENING INHIBITOR (RIN), which is a critical TF of fruit ripening^18–20^. In addition to tomato, in the other fleshy fruit species, lncRNAs were also reported to be the key regulators of fruit development and ripening. In strawberry, the lncRNA FRILAIR serves as miRNA sponge by functioning as a noncanonical target mimic of strawberry miR397, which can guide the mRNA cleavage of the fruit-ripening accelerating gene *LAC11a*, thereby regulating the fruit ripening process^21^. Due to the low conservation of lncRNAs from different species, it is difficult to predict lncRNAs function according to sequence similarity with reported lncRNAs. Therefore, genome-wide discovery and characterization of novel species-specific lncRNAs in fruits were conducted in various fleshy fruit species, including tomato^16,22^, strawberry^23^, sea buckthorn^24^, apple^25^, grape^26^, *Cucumis melo*^27^, *Prunus mume*^28^ and kiwifruit^29^.

Peach (*Prunus persica*) has become one of the most predominant temperate tree fruit species worldwide. Global peach (including nectarine) production increased from 5.2 million tones in 1961 to 25.7 million tones in 2019 according to Food and Agriculture Organization Statistics (FAOSTAT) 2021 data, and 61.5% of the peaches in 2019 were produced in China. However, more than twenty percent of the total production is lost at the postharvest stage due to the relatively short shelf life^30^. Peach is a typical climacteric fruit that dramatically increases the respiration rate during ripening, and the fruits deteriorate quickly at ambient temperatures during the postharvest stage^31^. To retard the respiration rate and extend the fruit shelf life, researches on regulatory networks of fruit ethylene biosynthesis, texture softening and cell senescence have been conducted in recent decades. Many TFs are involved in these processes, such as MADS-box, NAC, EIL and ERF TFs^20,32,33^. However, noncoding RNAs, especially lncRNAs, involved in fruit development and ripening processes remain largely elusive. In this study, we constructed 9 strand‑specific sequencing libraries using RNAs from three fruit developmental stages, the first exponential stage (S1), the second exponential stage (S3) and the fruit-ripening stage (S4). In total, 1500 confident lncRNAs (887 loci) were obtained using bioinformatics analysis. Combined with the transcriptome sequencing data, the characteristics, expressional features and candidate targets of lncRNAs in peach fruits were analysed. This study captured the landscape of lncRNAs in peach fruits, which will be helpful for further uncovering lncRNA members involved in fruit development and ripening processes.

## Results

### Genome‑wide identification of novel lncRNAs in different developmental stages of peach fruits

To comprehend the landscape and expression pattern of lncRNAs in peach fruits, 9 strand specific RNA-seq (ssRNA-Seq) libraries were constructed using the total RNA of peach fruits at 30, 49 and 65 days after full blooming (DAFB), representing the first exponential (characterized by cell division and primary enlargement), the second exponential (characterized by rapid cell enlargement) and the ripening stages, respectively, with three biological replicates for each. After trimming the adapters and low-quality reads, 157.17 Gb clean data were obtained, with Q30 > 93.69%. Then, the orientation of the reads and the genes were compared. More than 99% of the reads were mapped to the sense strand of the genes for each library (Table 1), further illustrating the strand-specific nature of our ssRNA-seq data. Subsequently, stand-specific data of high quality were used to search for lncRNAs.

**Table 1:** Summary of the ssRNA-Seq data of the peach fruits.

Library	Q30 (%)	Total reads	Mapped reads	Unique mapped reads (%)	Multiple mapped reads (%)	Read 1 vs gene	Read 2 vs gene
30 DAFB-1	94.23	126,387,374	95,952,800 (75.92%)	74.16	1.75	0.01	0.99
30 DAFB-2	93.69	128,342,020	109,994,234 (85.70%)	83.95	1.75	0.01	0.99
30 DAFB-3	93.87	118,638,866	101,493,991 (85.55%)	83.78	1.77	0.01	0.99
49 DAFB-1	94.31	120,145,242	96,904,692 (80.66%)	78.96	1.70	0.01	0.99
49 DAFB-2	95.03	105,238,944	85,861,264 (81.59%)	79.86	1.72	0.01	0.99
49 DAFB-3	94.72	111,798,776	93,800,478 (83.90%)	82.22	1.68	0.01	0.99
65 DAFB-1	94.63	109,489,338	94,624,947 (86.42%)	84.35	2.07	0.00	1
65 DAFB-2	95.06	108,784,612	93,188,392 (85.66%)	83.71	1.95	0.01	0.99
65 DAFB-3	94.55	138,062,940	117,453,929 (85.07%)	83.04	2.03	0.01	0.99

**Figure 1:**
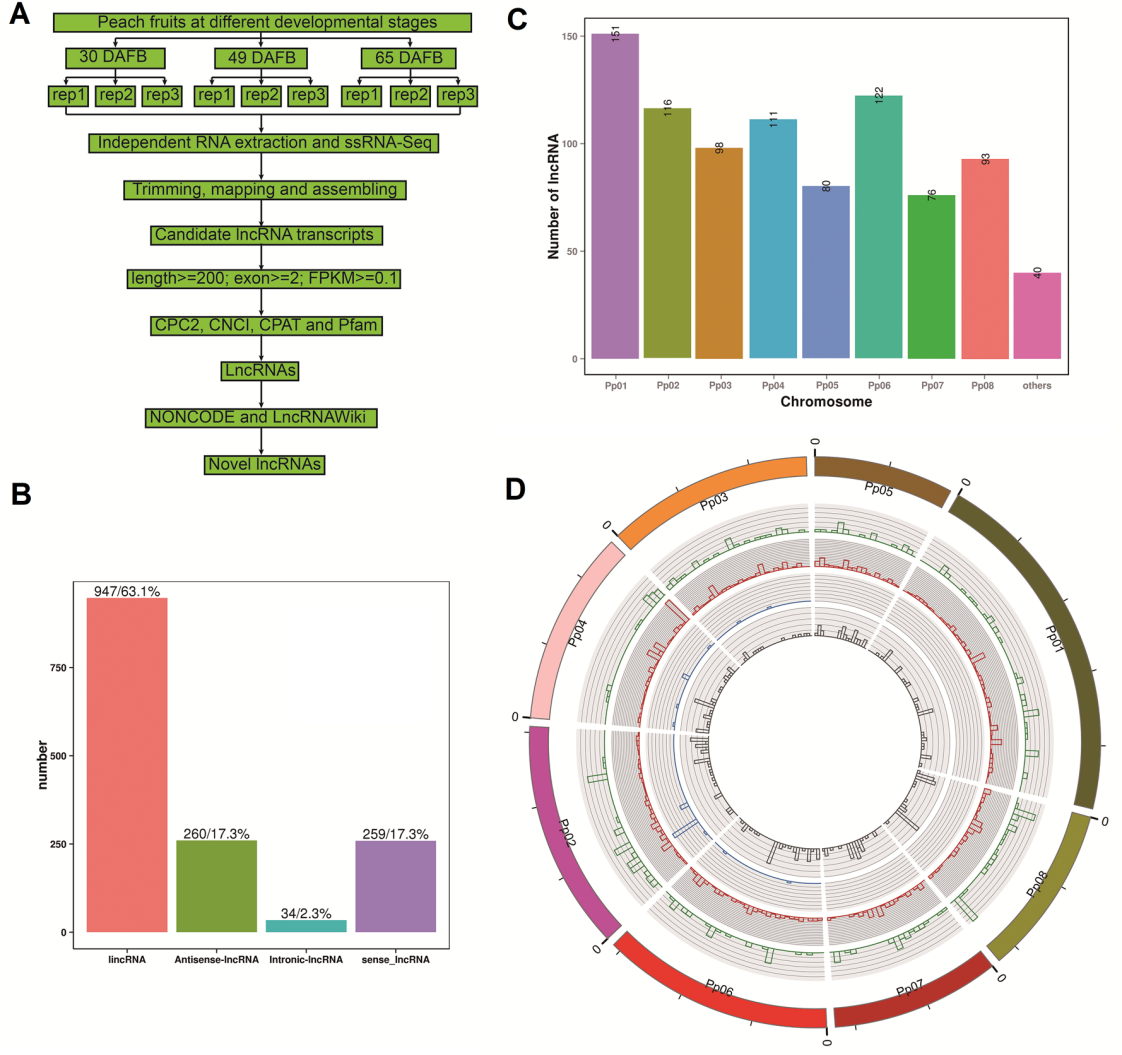
Characteristics of lncRNAs identified in peach fruits. (**A**) The pipeline used to identify novel lncRNAs from ssRNA-seq data. (**B**) Classification of identified lncRNAs based on the relative locations of lncRNAs and related genes. (**C**) Distribution of lncRNAs on different chromosomes. (**D**) Circos plot shows the overall distribution of four types of lncRNAs in the genome. Tracks from outside to inside are sense lncRNAs, lincRNAs, antisense lncRNAs and intronic lncRNAs.

For the 9 libraries, 75.9–86.4% of the clean reads were mapped to the peach genome v2.0^34^ reference sequences, and 74.2–84.4% were uniquely mapped (Table 1). Because no lncRNA annotation has been performed in peach fruit before, all mapped reads were further assembled using StringTie software (Fig. 1A). The assembled transcripts were then compared with the peach gene annotation file (gff) and were categorized into different classes, and only the transcripts annotated with symbols ‘u’, ‘i’, ‘o’ and ‘x’ were retained, which represent intergenic, intronic, sense and antisense transcripts, respectively (Fig. 1B). Then, transcripts with length < 200 without introns or with expression levels (FPKM) < 0.1 were removed from the candidate lncRNA database. For the last step, three methods CPC2 (Coding Potential Calculator), CNCI (Coding-Non-Coding Index) and CPAT (Coding Potential Assessment Tool) were adopted to predict the noncoding transcripts, and 3706, 1853 and 3686 putative noncoding transcripts were obtained, respectively (Fig. 1A). The Pfam database (34.0) was also used to eliminate the transcripts (446) with conserved domains with known coding genes (Fig. 1A). Finally, combining the results from the CPC2, CNCI, CPAT and Pfam databases, 1500 confident lncRNAs (887 loci, Table S1) comprising 947 lincRNAs, 260 antisense lncRNAs, 34 intronic lncRNAs and 259 sense lncRNAs, expressed in peach fruits were obtained (Fig. 1A, Table S1). Overall, for the 8 chromosomes, chromosome 1 had the largest number (151) of lncRNA loci, followed by chromosome 6 (122) and chromosome 2 (116), and the smallest number (76) was found on chromosome 7 (Fig. 1C).

The distribution of lncRNA transcripts identified in this study on the genome was illustrated by Circos plot (Fig. 1D). It is obvious that the lncRNAs identified in this study were not distributed uniformly in the genome. For example, intronic lncRNAs identified in this study were only present on chromosomes 2, 3, 4 and 6, but not on the other four chromosomes. Antisense-lncRNAs identified in this study were distributed on each part of chromosome 4, but sense-lncRNAs identified in this study were almost only present on the end but not the other part of chromosome 4. However, it should be noted that the lncRNAs identified in this study were only derived from fruits, therefore their distribution may be different from that of the overall lncRNAs of peach from various tissues.

To estimate the sequence conservation of lncRNAs at the inter-species level, the lncRNAs identified in this study were used as queries to blast against the NONCODE^35^ database to search for homologies from other species including *Arabidopsis*, *Brassica napus*, *Brassica rapa*, *Chenopodium quinoa*, cucumber, soybean, *Gossypium raimondii*, apple, cassava, *Medicago truncatula*, banana, *Oryza. rufipogon*, populus, tomato, potato, cacao, trefoil, wheat, grape and maize. Overall, 65 (4.3%) peach lncRNAs found homologues (evalue < 0.001) in the NONCODEV6 database; or in other words, 473 lncRNAs were homologous with peach lncRNAs in this study (Table S2). When searching for lncRNA homologues in a database of specific species, we found that no homologs were found in several species including soybean, rice, populus, tomato, cacao and trefoil (Table 2). Positional conservation analysis of the lncRNAs between peach and apple were also carried out, and the results showed that comparing to the extremely low sequence conservation ratio (0.4%), a high ratio 43.3% (651) of peach lncRNAs showed positional conservation with apple lncRNAs according to our criteria. These results indicated the peach lncRNAs shows high divergence at the nucleotide level, but high conservation by position.

**Table 2:** Identification of conserved lncRNAs in other plant species from the NONCODEv6 database.

Number of peach lncRNAs with homologs in other species	Conservation ratio of peach lncRNAs (%)	Total lncRNAs in other species	Other species
21/6	1.40	4046	*A*. *thaliana*
15/17	1.00	8212	*B*. *napus*
17/6	1.10	6457	*B*. *rapa*
31/36	2.00	9928	*C*. *quinoa*
11/7	0.07	2550	*C*. *sativus*
0/0	0.00	2242	*G*. *max*
12/3	0.80	1247	*G*. *raimondii*
6/12	0.40	1843	*Malus* × *domestica*
10/14	0.67	5601	*M*. *esculenta*
19/4	1.30	2258	*M*. *truncatula*
21/2	1.40	1809	*M*. *acuminata*
22/3	1.50	7616	*O*. *rufipogon*
0/0	0.00	1190	*O*. *sativa*
0/0	0.00	2248	*P*. *trichocarpa*
2/1	0.10	3822	*S*. *lycopersicum*
14/6	0.90	3069	*S*. *tuberosum*
0/0	0.00	3532	*T*. *cacao*
0/0	0.00	5278	*L*. *corniculatus*
23/344	1.50	12,427	*T*. *aestivum*
21/10	1.40	3351	*V*. *vinifera*
12/1	0.80	4717	*Z*. *mays*

### Comparing the features between lncRNAs and mRNAs

**Figure 2:**
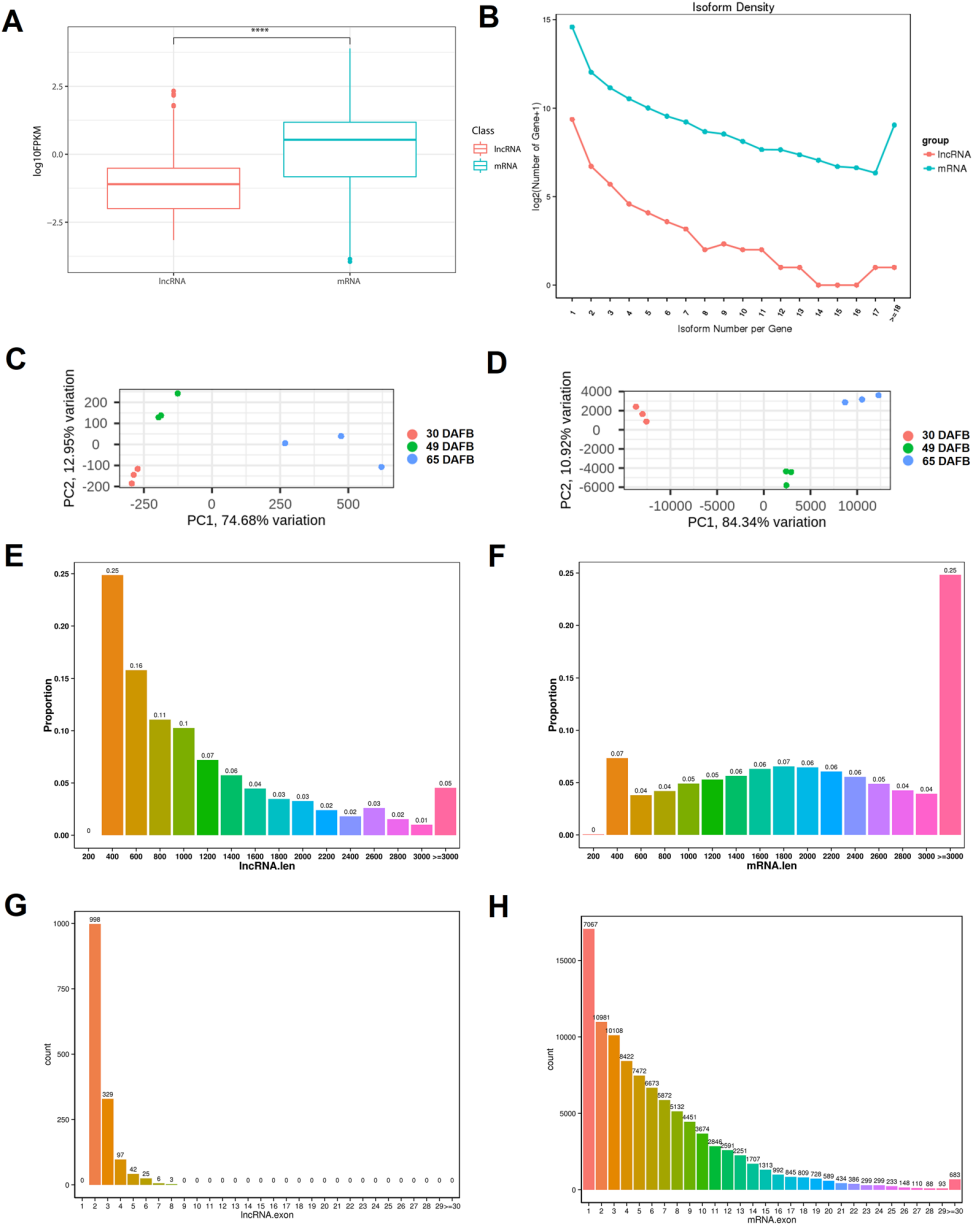
Comparison of the characteristics of lncRNAs and mRNAs. Overall analysis of expression levels (**A**), isoform complexity (**B**), PCA of gene expression levels of each sample (**C,D**), length distribution (**E**,**F**) and exon number analysis of lncRNAs and mRNAs (**G**,**H**). ****, *P* < 0.0001 (Student’s *t*-test).

The expression levels of lncRNAs and mRNAs were estimated using FPKM (fragments per kilobase of transcript per million fragments mapped). Overall, the expression levels of lncRNAs, were significant lower (*P* < 0.0001) than the levels at which protein-coding genes were expressed (Fig. 2A). Isoform numbers per lncRNAs was much less than that of mRNA (Fig. 2B). PCA showed that the expression data of both lncRNAs and mRNAs could distinguish the three developmental stages (Fig. 2C and D), indicating the fruit-developmental-stage-specific expression patterns of both lncRNAs and mRNAs. However, three biological replicates of lncRNA libraries showed dispersion at the ripening stage compared with the mRNA, which suggested a weaker constraint of lncRNA than mRNA during the fruit-ripening process.

Next, we analysed the length distribution and exon number between lncRNAs and mRNAs. Transcripts with lengths below 200 nt were removed when predicting the lncRNAs, and mRNAs with lengths less than 200 nt were not annotated as protein-coding genes (Fig. 2E and F). For lncRNAs, the length ranged from 202 to 10,356 nt, with a median length of 757 nt (Fig. 2E). However, 25% of mRNAs were larger than 3,000 nt, with a median length of 1,983 nt (Fig. 2F), which indicated that lncRNAs were shorter overall than mRNAs. Because transcripts without exons were removed during the prediction of lncRNAs, all lncRNAs in this study contained at least two exons. Structural analysis showed that lncRNAs with two exons accounted for the maximum proportion (66.5%) and only 7 lncRNAs had exon numbers equal to or greater than 7 (Fig. 2G). However, only 18.5% of the mRNAs contained one or two exons in peach (Fig. 2H). All these data indicated that lncRNAs generally had less complicated structures than mRNAs.

### Identification of differentially expressed lncRNAs (DELs)

**Figure 3:**
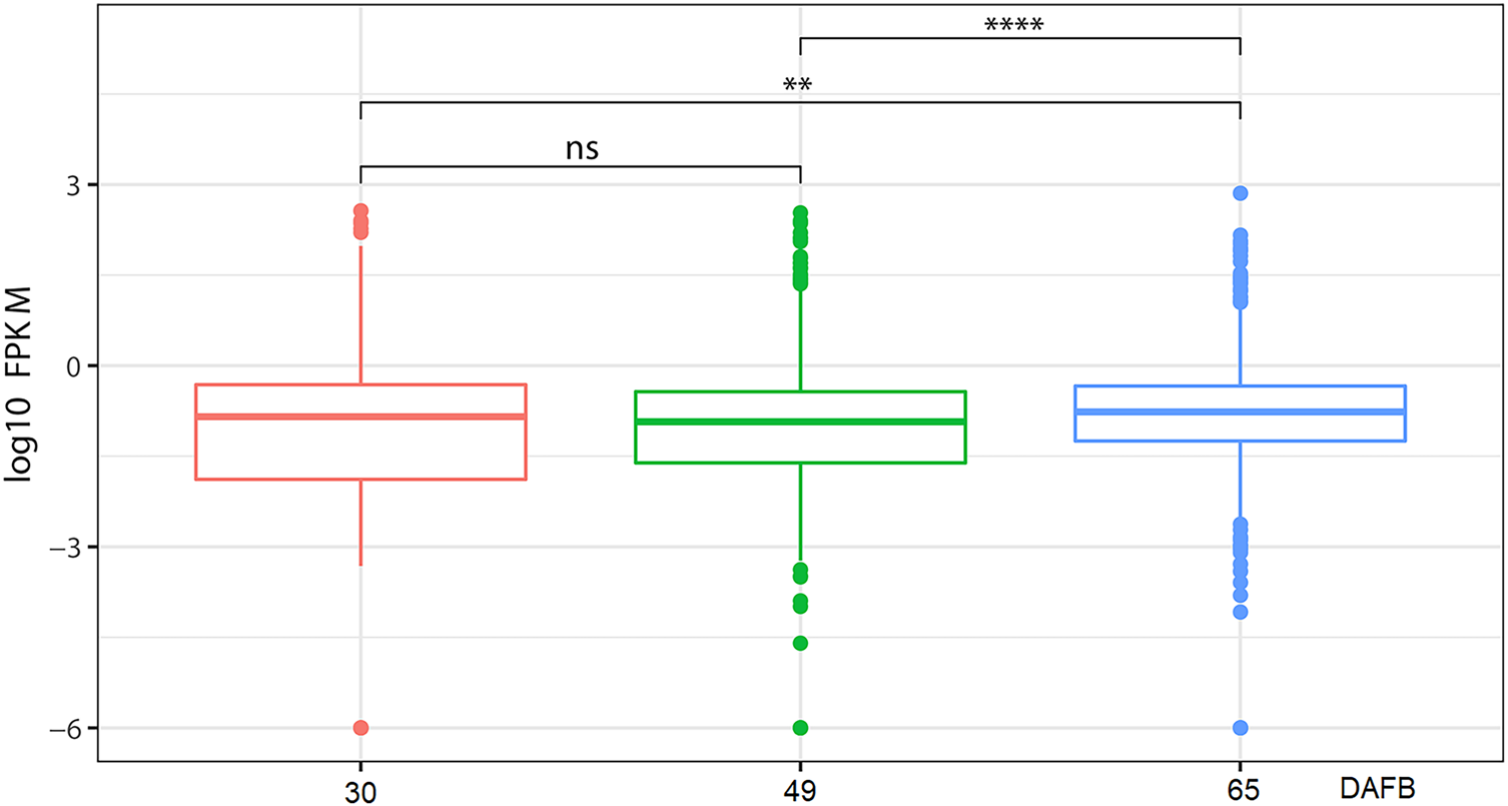
Overall expression levels of lncRNAs of each fruit developmental stage. ** and **** represent *P* < 0.01 and *P* < 0.0001 (Student’s *t*-test), respectively.

**Figure 4:**
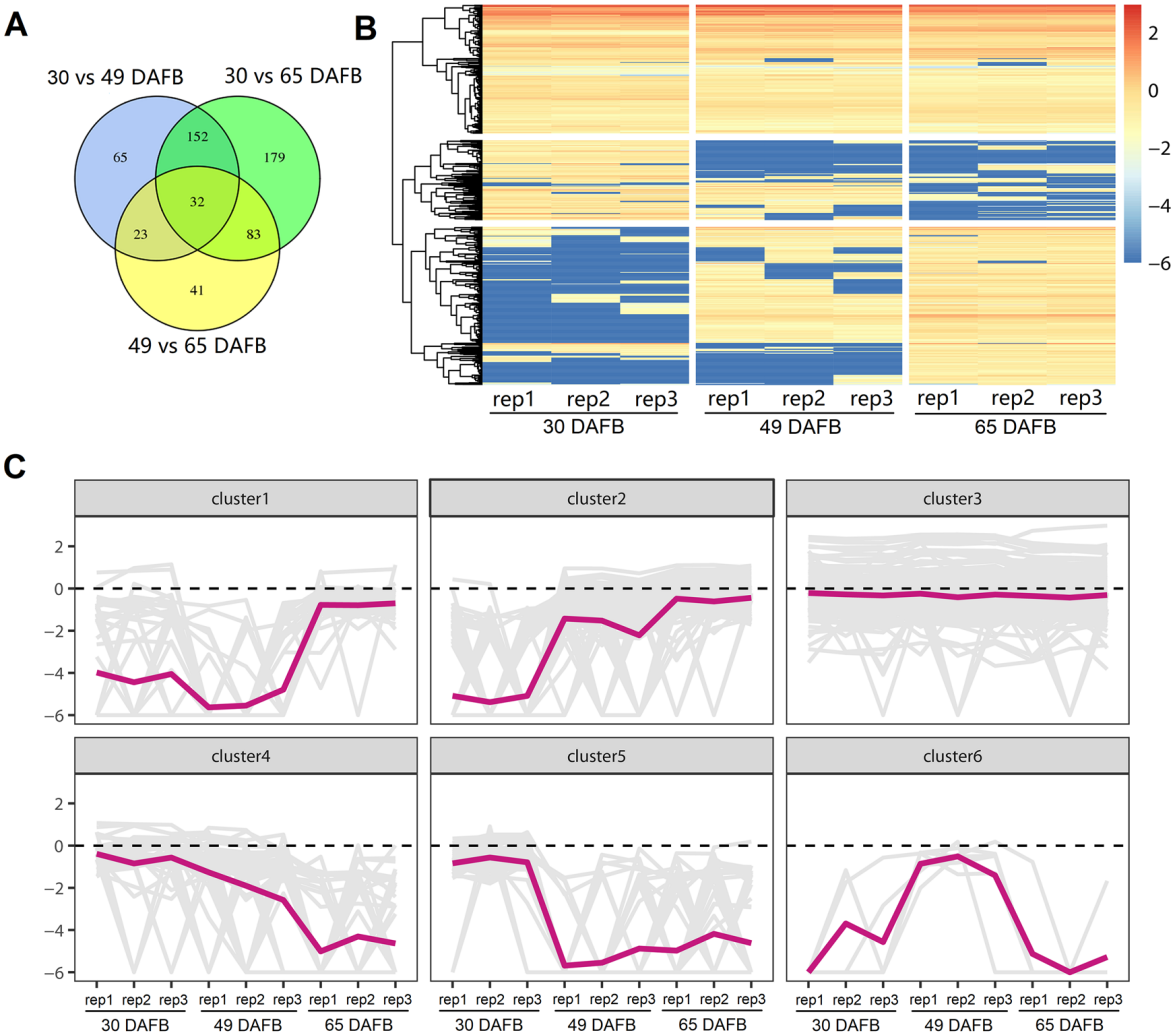
Overall view of the differentially expressed lncRNAs (DELs). Venn diagram (**A**), heatmap (**B**) and fitting curve (**C**) analysis of DELs.

The overall expression levels of lncRNAs were estimated for each library. As shown in Fig. 3, the median expression levels of lncRNAs at 49 DAFB were relatively lower than those at 30 and 65 DAFB. Results of statistical analysis showed that significant difference were found for overall lncRNA expression levels for ‘30 and 49 DAFB’ and ‘49 and 65 DAFB’. However, no significant difference was found between fruits at 30 and 49 DAFB (Fig. 3). Furthermore, the expression levels of lncRNAs were compared between groups, including ’30 vs 49 DAFB’, ‘30 vs 65 DAFB’ and ‘49 vs 65 DAFB’. In total, 575 DELs were obtained, with 272, 446 and 179 members for’30 vs 49 DAFB’, ‘30 vs 65 DAFB’ and ‘49 vs 65 DAFB’, respectively (Fig. 4A and Figure S1). A hierarchical cluster of the DELs showed that all 575 DELs were divided into 6 clustered groups depending on their expression levels in the 9 libraries (Fig. 4B). Fitting curves showed that DELs with expression levels of relatively low variation belonged to Cluster 3 (Fig. 4C). Clusters 5, 6 and 1 represented DELs showing high expressional bias at 30, 49 and 65 DAFB, respectively. DELs showing gradual upregulation or downregulation during fruit developmental stages were categorized into Clusters 2 and 4. To understand the relationship of lncRNAs and fruit ripening, the DELs in Clusters 1, 2 and 4 were further investigated.

**Figure 5:**
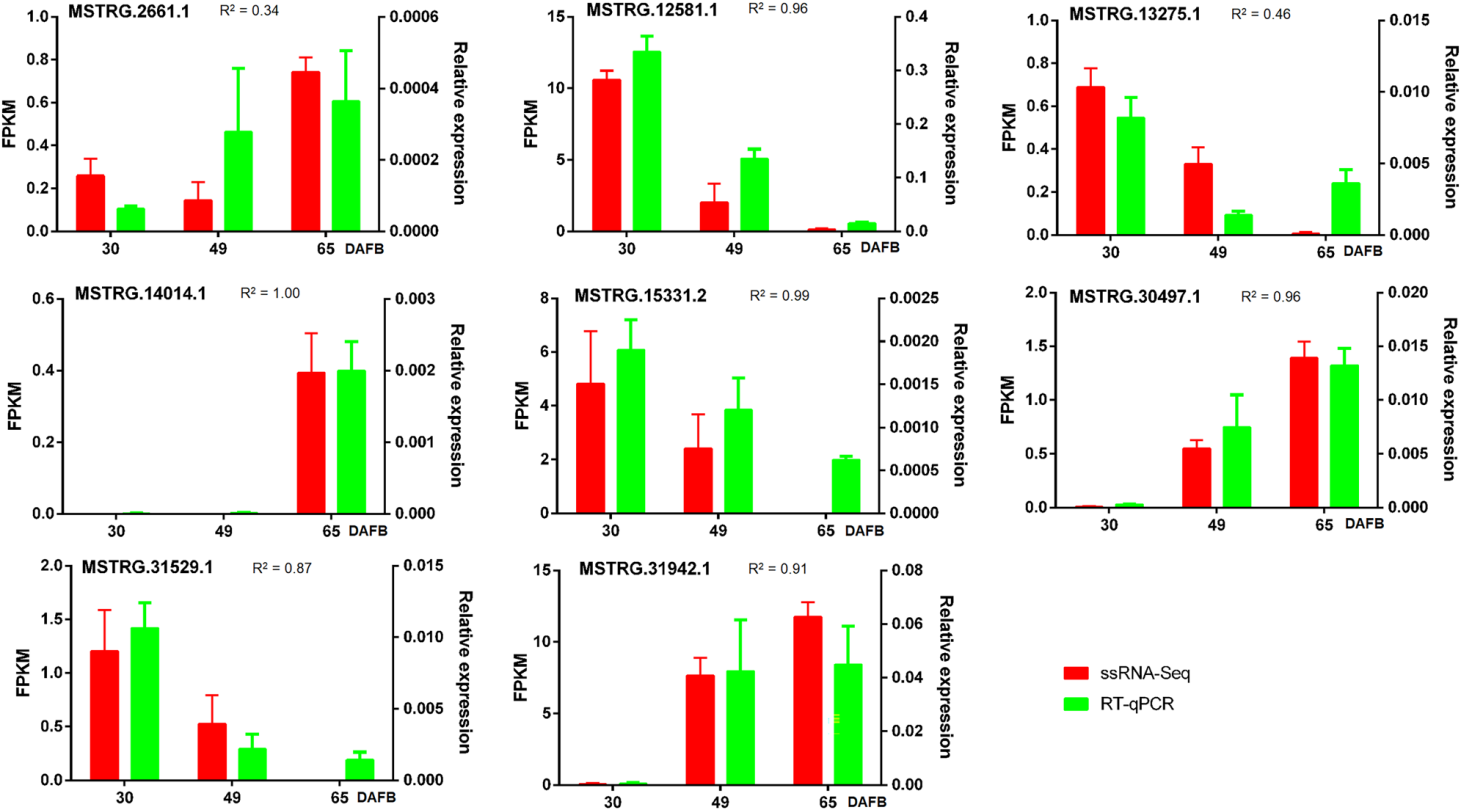
Expression profiles of randomly selected DELs by qRT–PCR and ssRNA-Seq. Error bars represent the SE of three biological replicates. R^2^, the correlation coefficients of Pearson correlation analyse.

Eight lncRNAs from Custers 1, 2, and 4 were randomly selected, and the expression levels of these lncRNAs in different developmental stages of peach cv. ‘Li Xia Hong’ were verified by RT–qPCR (Fig. 5). In general, most of the eight tested lncRNAs showed the same expression trends as the fitting curve clusters to which they belonged. For example, the two lncRNAs of Cluter 1, MSTRG.2662.1 and MSTRG.14014.1 both showed the highest average expression levels at 65 DAFB compared with 30 and 49 DAFB, although they showed different trends at 49 DAFB. Similarly, expression levels of MSTRG.20.497.1 and MSTRG.31942.1, two members of Cluster 2, both gradually increased during the developmental process. In contrast, for the remaining four Cluster 4 members, MSTRG.12581.1, MSTRG.13275.1, MSTRG.15331.2 and MSTRG.31529.1, all showed decreasing expression levels in adult fruits compared with the juvenile fruits. Moreover, Pearson correlation analyses revealed that the correlation coefficients (R^2^) between ssRNA-seq and RT–qPCR results were generally high (0.87–1.00 for 6 of the 8 tested lncRNAs), except for two of them, with R^2^ values 0.34 and 0.46 (Fig. 5). These results indicated that the RT–qPCR results were generally in line with the ssRNA-seq based quantifications of lncRNA levels.

### Functional annotation of the DELs

One of the ways lncRNAs function is by affecting the neighbor gene expression levels, and the affected genes are called cis-targets^36^. The genes located upstream or downstream of the lncRNAs were listed as candidate cis-targets. Following these criteria, 7,103 targets were obtained for 575 DELs, with an average of 12 candidate targets per DEL. Meanwhile, trans-targets were also predicted by screening the lncRNA-mRNA pairs depending on free energy of the interaction. In this way, 4,186 mRNAs were found to potentially interact with 575 DELs. To understand the possible functions of the DELs during fruit development and ripening processes, Gene Ontology (GO) and KEGG annotation and enrichment of the putative lncRNA cis- and trans- targets were performed.

**Figure 6:**
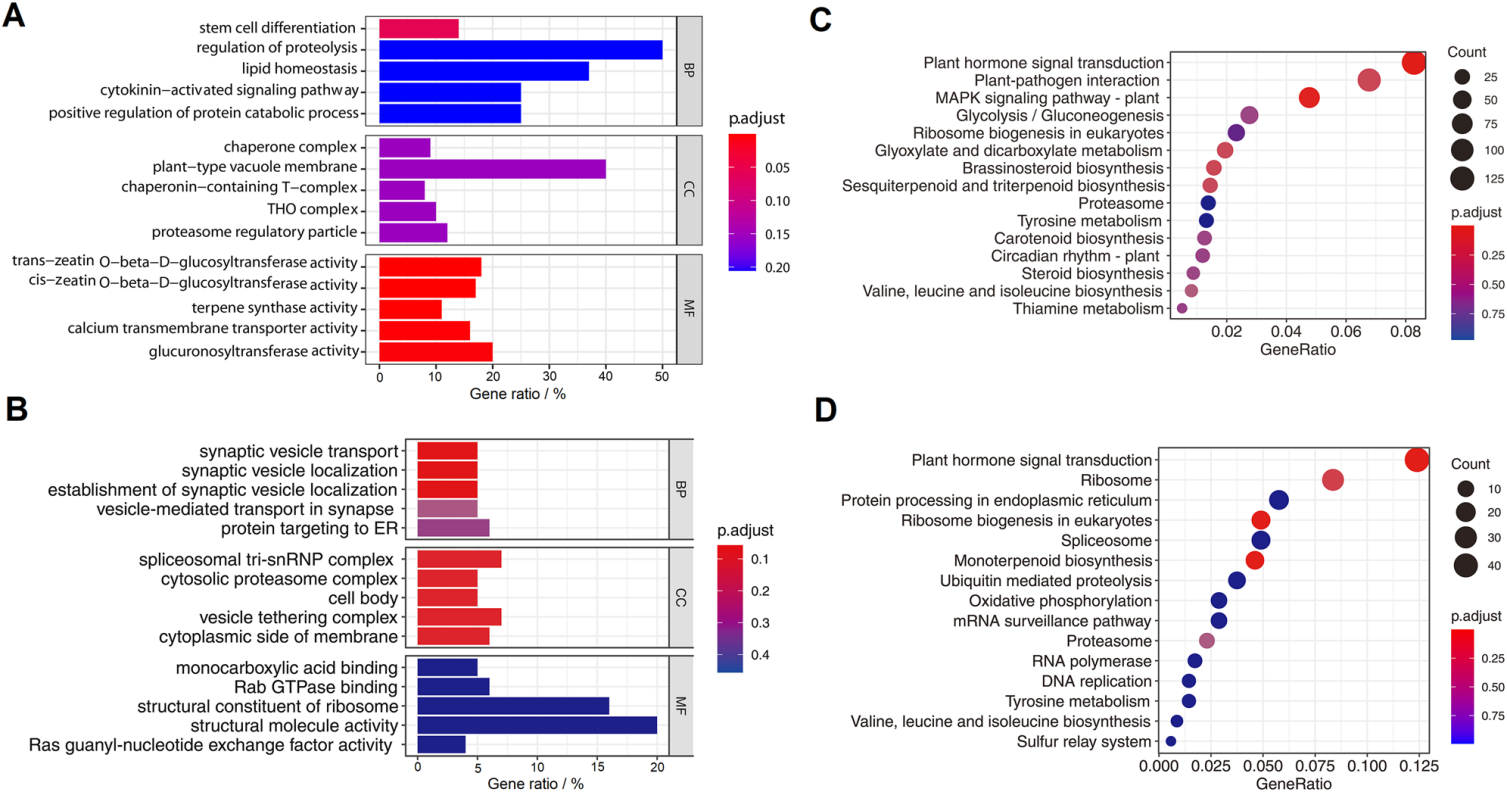
GO and KEGG annotation and enrichment of the putative targets of DELs. GO enrichment of cis- (**A**) and trans- (**B**) targets of DELs. BP, biological process. CC, cell component. MF, molecular function. KEGG enrichment of cis- (**C**) and trans- (**D**) targets of DELs.

As shown in Fig. 6A and B, GO terms of the cis- or trans-targets of DELs were enriched cross all GO categories of biological process (BP), cell component (CC) and molecular function (MF). Both the two kinds of targets showed enrichment in glycosyltransferase activities of MF (Fig. 6A and B). For cis-targets, enrichments of MF terms ‘trans-zeatin O-beta-D-glucosyltransferase activity’, ‘cis-zeatin O-beta-D-glucosyltransferase activity’, ‘terpene synthase activity’, ‘calcium transmembrane transporter activity’ and ‘glucuronosyltransferase activity’ had a statistically significant effect (Fig. 6A), and for trans-targets, ‘quercetin 3- or 7-O-glucosyltranserase activity’, ‘UDP-glucosyltranserase activity’, ‘UDP-glycosyltranserase activity’ and ‘glucosyltranserase activity’ were significantly enriched (Fig. 6B). However, the cis- or trans-targets of DELs showed great differences of enrichment in BP and CC categories. Comparing to the low adjusted P-values for cis-targets in BP and CC terms, trans-targets of DELs were significantly enriched for BP terms ‘RNA splicing’, ‘positive regulation of gene expression’ and ‘mRNA processing’ and CC terms ‘spliceosomal complex’, ‘nuclear speck’, and ‘thylakoid’ (Fig. 6A and B).

Similar with the GO enrichment results, tremendous differences of KEGG enrichment were also found between candidate cis- and trans-targets of DELs. For cis-targets, signaling transduction pathways such as ‘Plant hormone signal transduction’ and ‘MAPK signaling pathway’ were significantly enriched in cis-targets but not in trans-targets (Fig. 6C and D). On the contrary, several secondary metabolites synthesis pathways were enriched in trans-targets but not in cis-targets, such as ‘linoleic acid metabolism’, ‘Flavone and flavonol biosynthesis’ and ‘Anthocyanin biosynthesis’ (Fig. 6C and D). Moreover, pathways ‘Carotenoid biosynthesis’ and ‘Ribosome biosynthesis in eukaryotes’ were both enriched in cis- and trans-targets of DELs (Fig. 6C and D).

A previous report has showed that the metabolic characteristics of fruit growth and fruit ripening are different although they are similar in some physiological changes^37^. Although unlike the fierce physical and chemical changes occurring during the fruit ripening stage, obvious changes also occurs during fruit growth stages. From the first exponential stage to the second exponential stage, as the fruits expand, soluble sugars (fructose, fucose, glucose, maltose, etc.), organic acids (benzoate, citrate, glycerate, etc.) and pigments (anthocyanins or carotenoids) also accumulate^37–40^. When comparing the GO and KEGG annotations of candidate targets of the DELs, we found common and different features between fruit growth (30 to 49 DAFB) and ripening (49 to 65 DAFB) processes. GO enrichments results showed that cis-targets enrichments of fruit growth and ripening processes were totally different (Figure S2A and B). On the contrary, for trans-targets enrichments, several GO terms related to glycosyltransferase activities were both enriched in the two processes (Figure S2C and D), indicating the possible roles of the corresponding lncRNAs during fruit development and ripening. KEGG pathway enrichment showed that ‘plant hormone signal transduction’ was all abundantly enriched for cis-targets in both processes, implying the important roles of hormones related lncRNAs in fruit development and ripening (Figure S3A and B). Similarly, several KEGG pathways, such as ‘Spliceosome’, ‘Tryptophan metabolism’, and ‘2-Oxocarboxylic acid metabolism’ were all abundantly enriched for trans-targets in both processes (Figure S3C and D). However, ‘phenylpropanoid biosynthesis’ and ‘Flavone and flavonol biosynthesis’ were enriched in the ripening process but not the fruit growth process (Figure S3A and B), which was in accordance with the phenylpropanoid and flavonoid compounds changes during fruit ripening process. Moreover, it was interesting that ‘Fatty acid degradation’ and ‘Fatty acid biosynthesis’ were enriched in fruit growth and ripening processes, respectively (Figure S3A and B). This result was in accordance with the fatty acid changes during fruit phase transition.

**Figure 7:**
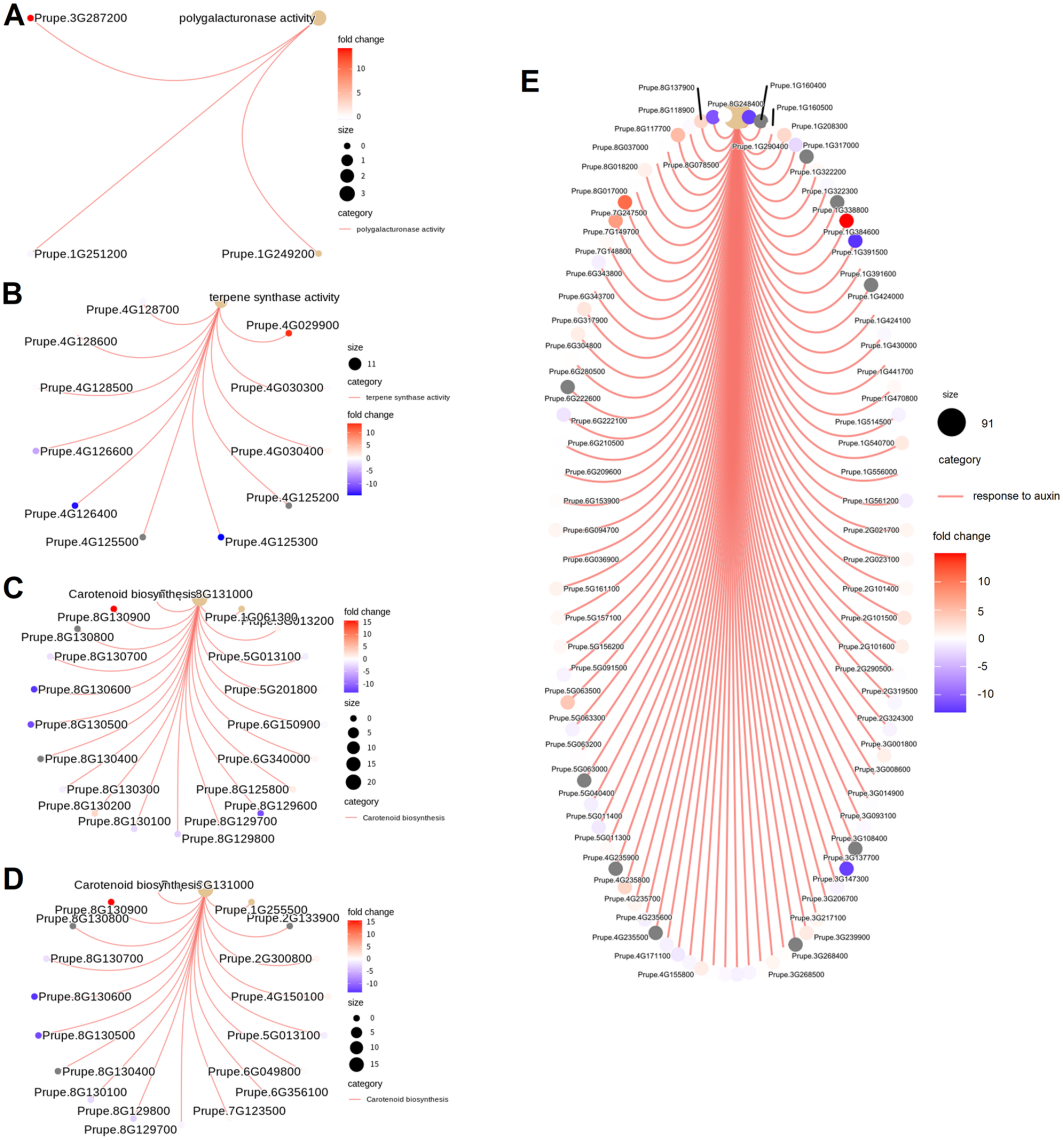
Cnetplot for various GO terms and KEGG pathways associated with putative targets of DELs. (**A**) cis-targets of DELs related to ‘polygalacturonase activity’. (**B**) cis-targets of DELs related to ‘terpene synthase activity’. Cis- (**C**) and trans-targets (**D**) of DELs related to ‘carotenoid biosynthesis’. (**E**) cis-targets of DELs related to ‘response to auxin’.

To further examination of the lncRNAs related to fruit ripening process, putative target genes of DELs related to ‘polygalacturonase activity’, ‘terpene synthase activity’, ‘carotenoid biosynthesis’ and ‘response to auxin’ were analyzed (Fig. 7). In total, 3 putative cis-target genes were annotated to be related to ‘polygalacturonase activity’, among which, a pectin lyase-like protein encoding gene (*Prupe.3G287200*) showed up-regulated during fruit ripening process (Fig. 7A). Likewise, 11 terpene synthesis related genes were enriched in cis-targets, among which, a terpene synthase gene (*Prupe.4G029900*) showed significantly higher expression level at 65 DAFB than at 49 DAFB (Fig. 7B). Moreover, it is interesting that two nine-cis-epoxycarotenoid dioxygenase encoding genes, *Prupe.1G061300* (a homologue of *AtNCED6*) and *Prupe.1G255500* (*PpCCD4,* the yellow-flesh peach determining gene^40^) were enriched in cis- and trans-targets of DELs, respectively and showed up-regulated during fruit ripening process (Fig. 7C and D). Finally, according to previous reports, auxin signal transduction plays critical roles in ethylene synthesis and fruit ripening in peach^41,42^. Indeed, multiple auxin-response genes were enriched as putative targets of the DELs between at 49 and 65 DAFB, and some of them showed significantly higher expression levels at the fruit ripening stage (Fig. 7E). These results demonstrate the possible involvement of lncRNAs in fruit developmental phase transitions.

### Computational identification of the ceRNA networks

**Figure 8:**
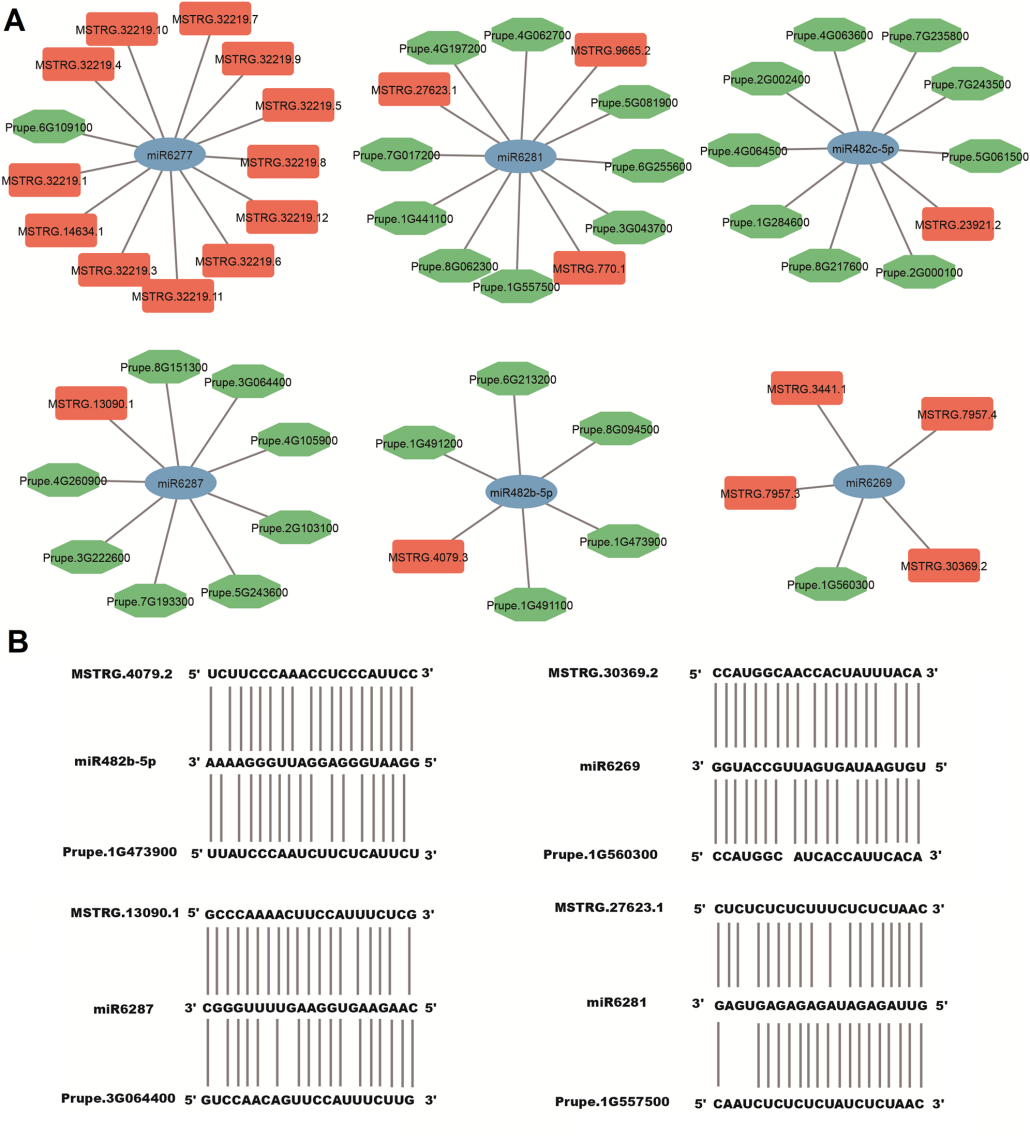
Competing endogenous RNA (CeRNA) networks of peach lncRNA-miRNA-mRNA. (**A**) Interaction networks of miRNA-lncRNA and miRNA-mRNA. (**B**) Sequence complementarity of lncRNA-miRNA-mRNA. Mature miRNA sequences and corresponding partial lncRNA or mRNA sequences were used for alignments.

To construct the ceRNA networks, the peach miRNA sequences were retrieved from the Rfam database. Totally, 26 miRNA-lncRNA and 255 miRNA-mRNA pairs were obtained, and the ceRNA networks were illustrated in Fig. 8A. The ceRNA networks were further verified by sequence alignments of relevant lncRNA, miRNA and mRNA sequences (Fig. 8B).

## Discussion

Recent study of peach pollen development has discovered 785 lncRNAs of pollen^43^, however, information of peach fruit lncRNAs remain elusive. In this study, we identified 1500 confident lncRNAs including 947 lincRNAs, 260 antisense-lncRNAs, 34 intronic-lncRNAs and 259 sense-lncRNAs from peach fruits using bioinformatics analysis. Previous genome-wide studies of lncRNAs in model plants identified 6510, 15,910 and 7655 lncRNAs in *Arabidopsis*^44^, rice^45^, and populus^46^. The fact that the number of lncRNAs identified in this study was much lower than that in *Arabidopsis*, rice and populus are likely due to three reasons. One reason is that the samples used for the detection of lncRNAs in the three species comprised multiple organs and tissues. For example, the total RNAs of entire seedlings of Arabidopsis were used for ssRNA-Seq library construction^44^, and the rice samples included flower buds, flowers, flag leaves and roots. The second reason may be due to the variation in lncRNAs distribution among different tissues. The fruit samples were usually enriched in fewer lncRNAs than the leaves. For instance, 9792 lncRNAs were identified in the leaves of tomato^47^, whereas only 1411 or 2508 lncRNAs were detected in tomato fruits^48,49^. Similarly, in three citrus species, trifoliate orange, pummelo and sweet orange, the numbers of lincRNAs in leaves were much higher than those in the other studied tissues, including fruits^50^. The final possible explanation is that the numbers of total lncRNAs vary in different species, even for the species in the same tribe. For example, when counting the lncRNA numbers in ovules, leaves, fruits and seeds, the citrus species Pummelo contained 2167 lncRNAs; however another citrus species *Atalantia* contained 3285 lncRNAs^50^.

According to previous reports in various plants, lncRNAs show distinct characteristics compared with mRNAs^2,8,13^. One feature is their relatively low expression level compared with protein-coding mRNAs. In this study, the expressional levels of lncRNAs, were 18- to 29-fold (median expression levels) lower than the levels at which protein-coding genes were expressed (Fig. 2A). These results are similar to the previous reports in regard to *Arabidopsis*^3,11^. Arabidopsis lincRNAs are expressed 30- to 60-fold lower than mRNA levels^11^, and the NAT expression level is similar to lincRNAs^3^. Nevertheless, a previous study in rice showed that the expression level of mRNAs is approximately 2.4- to 2.7-fold of that of lncRNAs^51^, indicating possible species variations of the overall expression level of lncRNAs. Another feature of lncRNA is their highly tissue-, organ- or developmental stage-specific characteristics. In this study, we found that approximately 24% of the lncRNAs showed developmental stage-specific expression patterns (Fig. 4A), which is similar to the proportion of tissue-specific lncRNAs (22%) in a previous research on tomato^52^.

LncRNAs usually show high divergence at the nucleotide level but higher conservation by position among species. In animals and plants, it has been reported that only 2–5.5% of lncRNAs are evolutionarily conserved in primary sequences^2^. Indeed, in this study, only 4.3% of the 1500 lncRNAs found in this study were homologous with those of the other species (Table S3), indicating a rapid evolutionary speed after peach speciation. It was reported that species of the same family still share low sequence conservation of lncRNAs. For example, only 5% and 6.7% homologous lncRNAs were found between rice and maize, and tomato and potato, respectively^52,53^. However, in our analysis, peach and apple seemed to share a very low proportion (0.40%) of homologous lncRNAs (Table 2), which was lower than that (1.40%) between peach and *Arabidopsis*. The low conservation of the lncRNAs between peach and apple found in this study may be due to the incomplete record of apple lncRNAs in the NONCODEv5 database. Compared with the lncRNA numbers in other species (e.g., 4046 in Arabidopsis, 8281 in *B. napus* and 12,427 in wheat), only 1843 apple lncRNAs were include in the database. When searching for the homologues in the apple genome (v3.0) using the 1500 peach lncRNAs as queries, it was shown that 20% of these peach lncRNAs found homologous hits in the apple genome (Table S4). The proportion is identical to the conservation proportion between rice lincRNAs and the maize genome (20%, approximately fourfold of the conservation proportion of lncRNAs between rice and maize), which further proves the deficient collection of apple lncRNA information to date^53^. However, the positional conservation analysis of peach and apple showed that 40% lncRNAs were conserved by position, which is higher than the positional conservation ration (25%) of *A. thaliana* and *A. lyrata*^54^. One explanation is that most of the investigated lncRNAs of peach and apple are from fruit tissues, which may increases the positional conservation ration due to the tissue-specific manner of lncRNAs. Therefore, to explore the sequence conservation lncRNAs, more lncRNA information from different tissues of species in the same family, genus or species needs to be obtained in the future.

The expression levels of lncRNAs are significantly affected by stress or developmental cues^4^. To date, studies on lncRNA responses to developmental cues have mainly been related to vegetation growth and floral development^8^. The study of the effects of lncRNAs on flesh fruit development and ripening is in its infancy, and has only been carried out on limited flesh fruit species, such as tomato and strawberry. In tomato, several fruit ripening related genes such as genes encoding ethylene synthesis or signalling enzymes (*ACO2*, *CTR1*), an ethylene responsive TF *ERF2* and three carotenoid accumulation enzyme-encoding genes (*PSY1*, *ZISO* and *NCED*) were predicted as the targets of several lncRNAs^55^. Furthermore, functional analysis of two tomato lncRNAs, lncRNA1459 and lncRNA1840, by CRISPR/Cas9-mediated mutagenesis and/or gene silencing revealed their essential functions in regulating fruit ripening^16,17^. In strawberry, genome-wide analysis also unearthed lncRNAs related to fruit development and ripening processes, especially anthocyanin accumulation^23,56,57^. In this study, several fruit development and ripening associated genes were also predicted as the targets of lncRNAs. For example, a gene encoding pectin lyase-like protein, possibly involved in cell wall remodeling, is potentially targeted by MSTRG.14836.3, and was up-regulated during fruit ripening process (Fig. 7A). Similarly, *PpTPS2*, a gene related to (E, E)-α-farnesene biosynthesis in peach^58^, is potentially targeted by MSTRG.15599.1, which showed increasing expression levels during fruit ripening (Fig. 7B). Recently, the strawberry lncRNA *FRILAIR* was found to derepress the fruit ripening related gene *LAC11a* by acting as a noncanonical target mimic of miR397^21^. In this study, several lncRNAs were also found to likely sequester miRNA with imperfect base complementarity (Fig. 8B). Our study provides valuable information on the lncRNAs involved in fruit development and ripening processes, whose function needs to be further validated by functional analysis in the future.

## Conclusion

In this study, we constructed ssRNA-Seq of 9 libraries from three different fruit developmental stages comprising the first and second exponential stages and the fruit ripening stage. After bioinformatics analysis, 1500 confident lncRNAs from 887 loci were obtained. The lncRNAs identified showed distinct characteristics compared with protein-coding mRNAs, such as lower expression level, lower complexity of alternative splicing, shorter length and smaller number of exons. Expression analysis of these lncRNAs showed that totally 575 DELs were obtained, with 272, 446 and 179 members for ‘30 vs 49 DAFB’, ‘30 vs 65 DAFB’ and ‘49 vs 65 DAFB’, respectively. The candidate targets of these lncRNAs were deduced by combining the location information and the putative interaction between DELs and protein-coding mRNAs. According to the GO and KEGG annotation of these DELs, the expression profiles and the ceRNA networks information, multiple lncRNAs potentially involved in the fruit development and ripening processes were predicted.

## Materials and methods

### Plant materials

The peach cultivar ‘Li Xia Hong’ was maintained in the experimental fields of the Anhui Academy of Agricultural Sciences, and the fruits were collected at 30, 49 and 65 DAFB representing the first exponential, the second exponential and ripening stages, respectively. After being peeled, cored and ground into powder, the fruit materials were immediately frozen in liquid nitrogen and stored at −80 °C until use. Three biological replicates were conducted for material collection, and each replicate contained at least 6 fruits.

### Library construction and strand‑specific sequencing

RNA was extracted using TRIzol methods, and RNA quality was assessed using 1.5% agarose gels (NanoDrop and Agilent Bioanalyzer 2100) to ensure the use of qualified samples for transcriptome sequencing. A total amount of 1.5 μg RNA per sample was used as input material for rRNA removal using the Ribo-Zero rRNA Removal Kit (Epicentre, Madison, WI, USA). Sequencing libraries were generated using NEBNext® Ultra™ II RNA Kits (NEB, USA) following the manufacturer’s instructions, and index codes were added to attribute sequences to each sample. Briefly, fragmentation was conducted using divalent cations under elevated temperature in NEBNext First Strand Synthesis Reaction Buffer (5X). First strand cDNA was synthesized using reverse transcriptase with random hexamer primers. Second-strand cDNA was synthesized using DNA Polymerase I and RNase H. The remaining overhang ends were blunted via exonuclease/polymerase activities. After adenylation of the 3’ ends of DNA fragments, NEBNext adaptors were ligated prior to hybridization. The library fragments were then purified with AMPure XP Beads (Beckman Coulter, Beverly, USA) to preferentially select insert fragments 150–200 bp in length. Then, size selection and adaptor ligation were performed using USER Enzyme (NEB, USA) before PCR. After PCR, the products were purified (AMPure XP system), and the library quality was assessed on an Agilent Bioanalyzer 2100. The index-coded samples were clustered on an acBot Cluster Generation System using TruSeq PE Cluster Kitv3-cBot-HS (Illumina) according to the manufacturer’s instructions. Afterwards, the library was sequenced on an Illumina HiSeq platform in paired-end read mode.

### Discovery and classification of novel lncRNAs in peach fruits

Raw data in fastq format were first processed through Perl scripts. In this step, clean data were obtained by removing adapter sequences and low-quality reads from the raw data. The strand-specific nature of the 9 libraries was evaluated using RSeQC software. All downstream analyses were based on strand-specific and clean data with high quality from this step.

The clean reads were mapped to the peach reference genome v2.0^34^ using Hisat2 (v 2.0.4)^59^. The mapped reads were then assembled into transcripts using StringTie (v1.3.1) with the parameter ‘-G’^60^. Then, gffcompare software was used to compare the assembled transcripts with the peach genome annotation (gff file) and obtain the unknown transcripts that were not annotated in the peach genome project. In this step, only the transcripts annotated with symbols ‘u’, ‘i’, ‘o’ and ‘x’ were retained, which represent intergenic, intronic, sense and antisense transcripts, respectively. Next, four computational approaches, CPC2^61^, CNCI^62^, Pfam^63^ and CPAT^64^, were combined to sort nonprotein-coding RNAs in the unknown transcripts, which were used to screen the lncRNAs. Finally, putative nonprotein-coding transcripts with lengths greater than 200 nt and at least two exons were selected as lncRNA candidates. The lncRNAs were categorized into different types of lncRNAs, including lincRNAs, intronic lncRNAs, antisense lncRNAs and sense lncRNAs, using cuffcompare v2.1.1 with default parameters. The distribution of these four types of lncRNAs on the eight chromosomes of the peach genome was plotted using Circos software.

### Searching for lncRNA homologues in the other species

Sequences of lncRNAs recorded in the NONCODEv6 database^35^ of different plant species (including *Arabidopsis*, *B. napus*, *B. rapa*, *C. quinoa*, *C. reinhardtii*, cucumber, soybean, *G. raimondii*, apple, cassava, *M. truncatula*, *O. rufipogon*, *P. trichocarpa*, tomato, potato, cacao, trefoil, wheat, grape and maize) were retrieved and used to build blast databases. The lncRNA homologues in these species were identified by the blastn program with the E-value < 0.001 and coverage ≥ 20%. Positional conservation analysis was carried out according to a previous report^54^ with small modifications. In brief, the region of nearest upstream and downstream protein coding genes of lncRNAs was used to assess collinearity among those selected species. Syntenic blocks between genomes of peach (v2.0) and apple (v1.0) were identified using MCScanX^65^. For each lncRNA analyzed, five protein-coding neighbor genes upstream and downstream were extracted, which were used to compare with the syntenic blocks. If four or more counterpart orthologous protein-coding neighbor genes were found in the same syntenic block, the corresponding lncRNAs from peach and apple were considered to be conserved by position.

### Expressional analysis of lncRNA and mRNA

Gene expression levels (FPKM, fragments per kilobase of transcript per million fragments mapped) of both lncRNAs and coding genes in each sample were calculated using StringTie (v1.3.1). PCA was calculated based on the expression levels of mRNAs and lncRNAs using the R package ‘PCAtools’. Differentially expressed genes of the two groups were analysed using the DESeq2^66^ R package (1.10.1). DESeq2 provides statistical routines for determining DEGs using a model based on the negative binomial distribution. The resulting *p* values were adjusted using Benjamini and Hochberg’s approach for controlling the false discovery rate. Genes or lncRNAs with an adjusted *P* value < 0.05 and absolute value of fold change > 2 found by DESeq2 were assigned as DEGs or DELs. DELs clustering and trend analyses were conducted using the R packages ‘ggplot2’, ‘pheatmap’ and ‘reshape2’.

### Prediction and annotation of peach lncRNA targets

Two bioinformatics methods were adopted to predict the lncRNA targets. First, the adjacent genes within the range of 100 kb of lncRNA were predicted as the cis-targets. Second, RIblast software was employed to calculate the annealing potential of the lncRNAs and mRNAs, and the mRNAs with a cutoff of interaction energy < −20 kcal/mol were predicted as the trans-targets. These candidate targets were annotated according to the GO (http://geneontology.org/) and KEGG (http://www.genome.jp/kegg/) databases using the eggNOG-mapper software (version v2, http://eggnog-mapper.embl.de/). GO and KEGG enrichments were conducted using R package ‘clusterProfiler’ 4.0^67^.

### Construction of ceRNA networks

Peach mature miRNA sequences were retrieved from Rfam (14.2) database (ftp://ftp.ebi.ac.uk/pub/databases/Rfam). Peach miRNA target searching at each mRNA and lncRNA sites was conducted using TarHunterL (v1.0) software^68^ with default parameters. CeRNA networks including candidate miRNA-lnRNA and miRNA-mRNA pairs were constructed using Cytoscape (3.9.1) software (https://cytoscape.org/).

### Quantitative real-time PCR (qRT–PCR)

Total RNA extraction was conducted using the RNAprep Pure Plant Kit (Polysaccharides & Polyphenolics-rich, TianGen, Beijing). Removal of DNA contamination and first strand cDNA synthesis were performed using the PrimeScript™ RT reagent Kit with gDNA Eraser (Takara Bio, Dalian). qRT–PCR of lncRNAs was conducted using TB Green® Premix Ex Taq™ (Tli RNaseH Plus, Takara Bio, Dalian.), with the following program: one cycle of 30 s at 95 °C for denaturation, followed by 40 cycles of 5 s at 95 °C and 30 s at 60 °C. After the amplification cycles, melting curves were obtained in all qPCR amplifications. *PpTEF2* gene was set as the internal reference gene for qRT–PCR. Three independent biological replicates were performed for each sample. Sequences of the qRT–PCR primers used in this study are listed in Table S4.

## Supplementary Information

Supplementary Information 1.

Supplementary Information 2.

Supplementary Information 3.

Supplementary Information 4.

Supplementary Information 5.

Supplementary Information 6.

Supplementary Information 7.

## Data Availability

The sequence data involved in this study have been deposited in NCBI SRA database (https://www.ncbi.nlm.nih.gov/sra/) with the Bio-Project accession number: PRJNA784390.
