# Supplementary material for: Genome-wide identification and characterization of long noncoding RNAs during peach (Prunus persica) fruit development and ripening
Source: Sci Rep. 2022 Jun 30;12:11044. doi: 10.1038/s41598-022-15330-3 (PMC9247041; doi:10.1038/s41598-022-15330-3)
Supplement: Supplementary file 1 — Supplementary Information 1. [file 41598_2022_15330_MOESM1_ESM.doc]

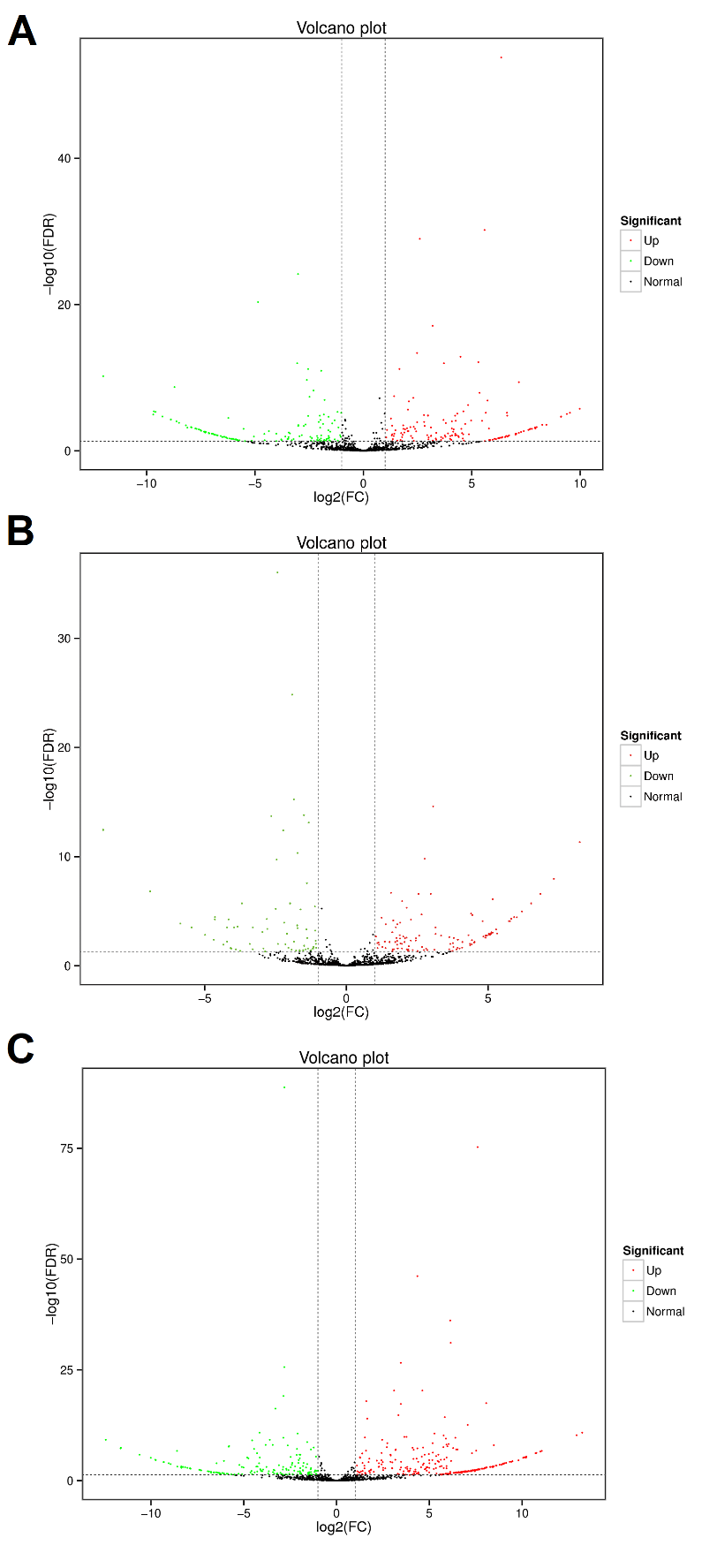


Figure S1 Volcano plots of the DELs during fruit development phase transitions. A, 30 vs. 49 DAFB; B, 49 vs. 65 DAFB; C, 30 vs. 65 DAFB.
