# Supplementary material for: Genome-wide identification and characterization of long noncoding RNAs during peach (Prunus persica) fruit development and ripening
Source: Sci Rep. 2022 Jun 30;12:11044. doi: 10.1038/s41598-022-15330-3 (PMC9247041; doi:10.1038/s41598-022-15330-3)
Supplement: Supplementary file 3 — Supplementary Information 3. [file 41598_2022_15330_MOESM3_ESM.docx]

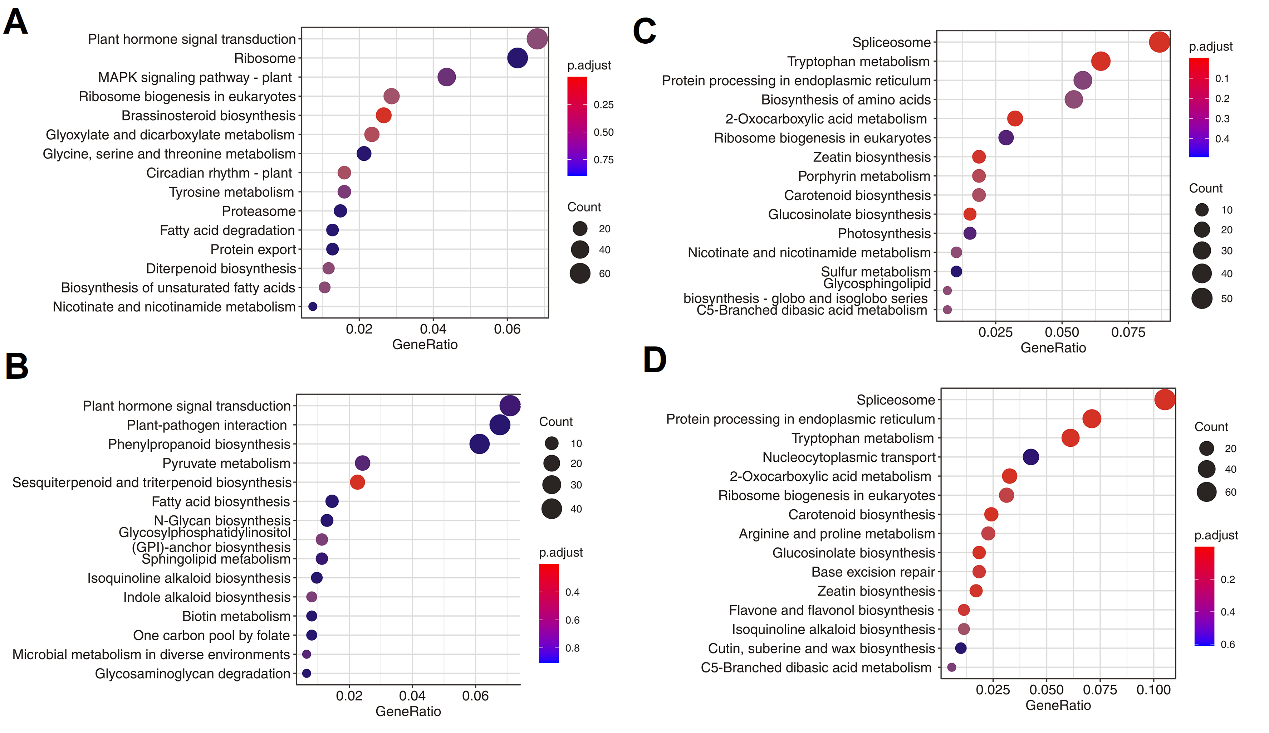


Figure S3 KEGG annotation and enrichment of the putative targets of DELs of fruit phase transitions. (A) cis-targets of ‘30 vs. 49 DAFB’. (B) cis-targets of ‘49 vs. 65 DAFB’. (C) trans-targets of ‘30 vs. 49 DAFB’. (D) trans-targets of ‘49 vs. 65 DAFB’.
