# Supplementary material for: Genome-wide identification and characterization of long noncoding RNAs during peach (Prunus persica) fruit development and ripening
Source: Sci Rep. 2022 Jun 30;12:11044. doi: 10.1038/s41598-022-15330-3 (PMC9247041; doi:10.1038/s41598-022-15330-3)
Supplement: Supplementary file 7 — Supplementary Information 7. [file 41598_2022_15330_MOESM7_ESM.doc]

Table S4. Sequences of primers used for qRT-PCR

| Primer name | Sequence | Description |
| --- | --- | --- |
| Q1F | GCCACCAAATTCGACGAGC | primers used for qRT-PCR of MSTRG.2661.1 |
| Q1R | CGATGCCAGGCCCACTTACT |  |
| Q2F | CATATGCTGAAAGCACTTCCAAT | primers used for qRT-PCR of MSTRG.14014.1 |
| Q2R | CATTAACAAACCAAATCACTCCCTA |  |
| Q3F | TGTTTATCTTCTTCTTGGCTCTGA | primers used for qRT-PCR of MSTRG.31942.1 |
| Q3R | GATATGCTTTCTTCTTCCAATTCC |  |
| Q4F | GCAGACTTTGAAACTTTGATTGG | primers used for qRT-PCR of MSTRG.30497.1 |
| Q4R | TCCTTTATTAGCTGGGTTTGGTT |  |
| Q5F | AAAGCAGCGCCGTCACAT | primers used for qRT-PCR of MSTRG.12581.1 |
| Q5R | TTCTTGGACGATGGAGGGTAA |  |
| Q6F | CACAACCCAACATTAGGCATCTC | primers used for qRT-PCR of MSTRG.31529.1 |
| Q6R | TGATGGCAAAGGGTGGAGAC |  |
| Q7F | CTTTCGGGACTGATTGGCTT | primers used for qRT-PCR of MSTRG.15331.2 |
| Q7R | GGGAAATAATCGGATGATAATGG |  |
| Q8F | GGGCTCCTCTACCTCCATCAT | primers used for qRT-PCR of MSTRG.13275.1 |
| Q8R | GTACAAGCGCAAGTTATCAGCATA |  |
|  |  |  |
